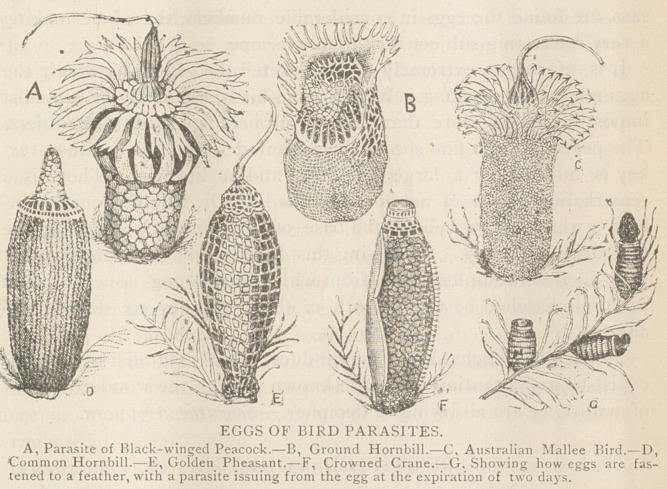# Eggs of Bird Parasites

**Published:** 1881-10

**Authors:** 


### POPULAR SCIENCE DEPARTMENT.

### EGGS OF BIRD PARASITES.

Among the little bird parasites are to be found the most extraordinary and fantastic structures.

The eggs of one of the species which infest the ground hornbill so much resemble the cells of some of the polyzoa that, deposited as they are in close contact one above another, and in many parallel lines between the flattened barbs on the inner surface of the feathers, they appear like some new species of sea-mat.

The strangely formed eggs found on the Australian crane are arranged in a similar manner, and a slide containing several rows of these eggs is a fine sight under the microscope.

EGGS OF BIRD PARASITES.

A, Parasite of Black-winged Peacock.—B, Ground Hornbill.—C, Australian Mallee Bird.—D, Common Hornbill.—E, Golden Pheasant.—F, Crowned Crane.—G, Showing how eggs are fastened to a feather, with a parasite issuing from the egg at the expiration of two days.

On one species of crowned crane (Balearica) are found eggs having a thick calcareous wall, being covered, as it were, with little white domes. Each of these projections appears to be deposited around and supported by a short spine proceeding from the shell of the egg, and supported by a sub-quadrate, pellate disk.

The egg of a parasite of the Australian mallee bird resembles somewhat the ripe fruit of the corn blue-bottle flower. The spines on the lowest or outer row on its summit are ornamented by little anchors, very like those of the Spicula synapia.

All these interesting eggs are, however, altogether exceeded in beauty by those of the Indian black-winged peacock, which are constructed so much like flowers that a botanist might amuse himself by describing every part of them in the technical language of his science.

The manner in which these eggs are deposited is also most singular. The animal attaches a mass of amorphous secretion to the inner side of the shaft of a feather, and then proceeds to construct two or thre oval perforate or punctuate sacs, much larger than the eggs. On and about, and in some cases buried, in these strange sacs, are found the eggs in considerable numbers, the whole making a very interesting subject for the microscope.

It is, of course, extremely difficult to tell the genera to which the eggs respectively belong. With foreign birds, especially, it is almost impossible to do more than form a probable guess on the subject. The peacock has a fine specimen of goniodes, and the common turkey is infested by a large goniodes and a lipeurus. There is a remarkable species of acarus, described by Dr. Robins, found spinning a white silken web on the base of the sparrow's thigh, or the forepart of its body. On raising this delicate web you perceive that it is filled with minute eggs, from which the young issue, being in due time hatched by the warmth of the body they are destined to annoy.

Perhaps this slight sketch may induce some naturalist or microscopist to pay attention to a little known page in the wonderful book of nature we are all trying to decipher.—*Scientific American.*

## Figures and Tables

**Figure f1:**